# Adherence to the World Cancer Research Fund/American Institute for Cancer Research recommendations for cancer prevention is associated with better health–related quality of life among long-term colorectal cancer survivors: results of the PROFILES registry

**DOI:** 10.1007/s00520-019-04735-y

**Published:** 2019-03-29

**Authors:** Merel R. van Veen, Floortje Mols, Martijn J. L. Bours, Matty P. Weijenberg, Ellen Kampman, Sandra Beijer

**Affiliations:** 1 Department of Research & Development, Netherlands Comprehensive Cancer Organisation (IKNL), IKNL, P.O. Box 19079, 3501 DB Utrecht, The Netherlands; 2 Division of Human Nutrition and Health, Wageningen University, P.O. Box 17, 6700 AA Wageningen, The Netherlands; 3 CoRPS-Center of Research on Psychology in Somatic diseases, Department of Medical and Clinical Psychology, Tilburg University, PO Box 90153, 5000 LE Tilburg, The Netherlands; 4 Department of Epidemiology, GROW – School for Oncology and Developmental Biology, Maastricht University, P.O. Box 616, 6200 MD Maastricht, The Netherlands

**Keywords:** Colorectal cancer survivors, Health-related quality of life, WCRF guidelines, Dietary guidelines, Physical activity, Body composition

## Abstract

Since colorectal cancer (CRC) survivors often suffer from long-term adverse health effects of the cancer and its treatment, having a negative impact on their health-related quality of life (HRQL), this study focuses on the association between adherence to WCRF/AICR recommendations and HRQL among CRC survivors. In a cross-sectional PROFILES registry study in 1096 CRC survivors (mean time since diagnosis 8.1 years), WCRF/AICR adherence scores (range 0–8, with a higher score for better adherence) were calculated, and HRQL was assessed using the EORTC QLQ-C30. Associations between adherence scores and HRQL scores were investigated using linear regression analyses. Additionally, associations with adherence to guidelines for body mass index (BMI) (normal weight, overweight and obese), physical activity (PA) (score 0/1) and diet (score < 3, 3– < 4 and > 4) were evaluated separately. Mean adherence score was 4.81 ± 1.04. Higher WCRF/AICR scores were associated with better global health status (*β* 1.64; 95%CI 0.69/2.59), physical functioning (*β* 2.71; 95%CI 1.73/3.68), role functioning (*β* 2.87; 95%CI 1.53/4.21), cognitive functioning (*β* 1.25; 95%CI 0.19/2.32), social functioning (*β* 2.01; 95%CI 0.85/3.16) and fatigue (*β* − 2.81; 95%CI − 4.02/− 1.60). Adherence versus non-adherence PA was significantly associated with better physical, role, emotional and social functioning, global health status and less fatigue. Except for the association between being obese and physical functioning (*β* − 4.15; 95%CI − 47.16/− 1.15), no statistically significant associations with physical functioning were observed comparing adherence to non-adherence to BMI and dietary recommendations. Better adherence to the WCRF/AICR recommendations was positively associated with global health status, most functioning scales and less fatigue among CRC survivors. PA seemed to be the main contributor.

## Introduction

In 2007, the World Cancer Research Fund/American Institute for Cancer Research (WCRF/AICR) launched the diet and physical activity recommendations for cancer prevention [1]. Cancer survivors, defined as people who are living with a diagnosis of cancer, including those who have recovered from the disease [1], or in other words those who finished treatment and are disease-free, are encouraged to follow these recommendations to reduce risk of recurrence and improve survival.

Colorectal cancer (CRC) survivors often suffer from long-term adverse health effects of cancer and its treatment [2]. This can have a negative impact on health-related quality of life (HRQL). Two systematic reviews showed that CRC survivors had a lower physical functioning and more fatigue and psychological problems, including depression, anxiety and distress than the general population [3, 4]. Because of the increasing numbers of CRC survivors, investigating possibilities to increase HRQL is very important.

Several studies showed an association between adherence to general non-cancer-specific dietary guidelines, such as the Healthy Eating Index or the Mediterranean diet, and higher levels of HRQL in cancer survivors, including CRC survivors [5–7]. In addition, previous studies have demonstrated that CRC survivors who met the public health exercise guidelines reported better quality of life (QL) and fatigue scores than CRC survivors who did not meet these guidelines [8, 9]. Although adherence to general dietary or exercise guidelines showed positive associations with HRQL, the association between adherence to the cancer-specific WCRF/AICR recommendations on diet, physical activity and body weight/composition and HRQL have only been investigated in female cancer survivors in general [10], in breast cancer survivors [11] and in a small cross-sectional study (*N* = 145) in CRC survivors [12]. These studies showed that better adherence to the WCRF/AICR recommendations was associated with better HRQL [10–12]. However, due to the relatively small numbers in these studies, it was not possible to evaluate which specific recommendations had the highest impact among CRC survivors (diet, physical activity or body composition). The aim of the present study was to investigate the association between adherence to the WCRF/AICR recommendations and HRQL for all recommendations together and for physical activity, body composition and diet separately in a large cohort of CRC survivors.

## Subjects and methods

### Study design

This study was part of an ongoing longitudinal study investigating HRQL in CRC patients. All CRC patients stage I–IV, diagnosed between January 2000 and June 2009 from the southern area of the Netherlands, were sampled via the Netherlands Cancer Registry (NCR). The Patient Reported Outcomes Following Initial Treatment and Long-term Evaluation of Survivorship (PROFILES) registry was used to collect the data [13].

Ethical approval for the study was obtained from the local certified Medical Ethics Committee of the Maxima Medical Centre Veldhoven, the Netherlands (approval number 0822). All participants gave informed consent. Data from this longitudinal study are (partly) available online for non-commercial scientific research, subject to study question, privacy and confidentiality restrictions, and registration (www.profilesregistry.nl).

### Data collection

CRC patients were invited for participation via a letter from their (former) attending physician. The letter included a link to a secure website, a login name and a password, so that interested patients could provide consent and complete questionnaires online. Those who preferred written communication could return a postcard after which they received our paper-and-pencil informed consent form and questionnaire. Non-respondents were sent a reminder letter and paper-and-pencil questionnaire within 2 months. Patients were reassured that nonparticipation had no consequences for their follow-up care or treatment. The NCR provided information on cancer diagnosis and cancer treatment history, such as year of diagnosis, stage and localization of cancer and having a stoma.

### Study population

**Fig. 1:**
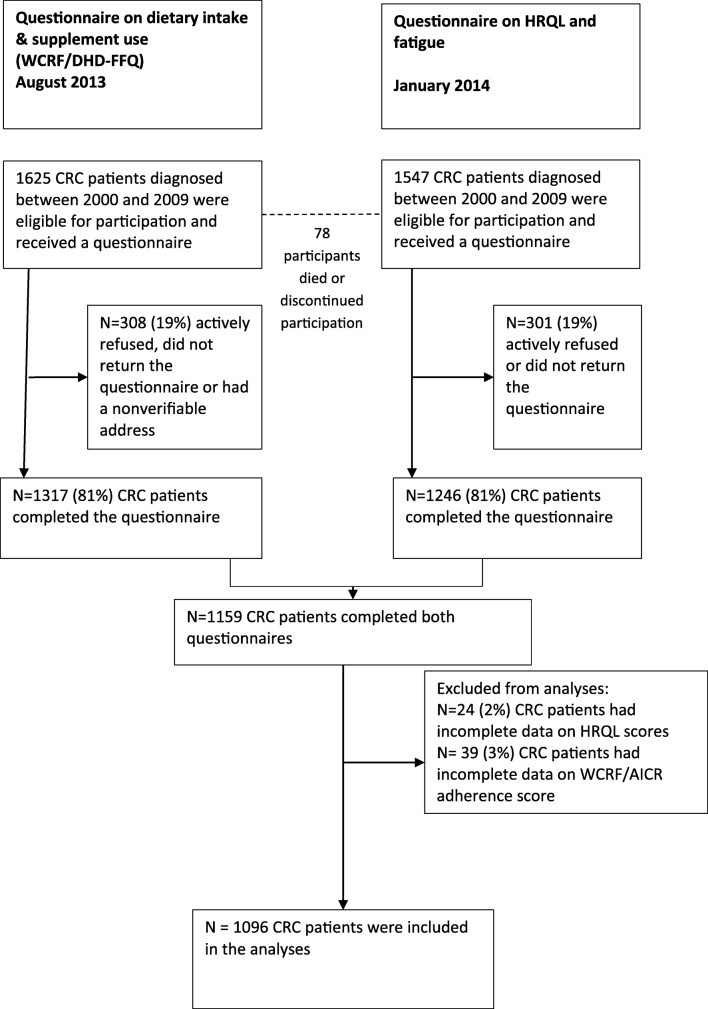
Flowchart of the study population

The CRC study started in December 2010 and respondents received subsequent HRQL questionnaires in December 2011, December 2012 and January 2014. In August 2013, data on the adherence to WCRF/AICR recommendations were collected once. A complete overview of the selection of patients can be found on our website under ‘data & documentation’; https://www.dataarchive.profilesregistry.nl/study_units/view/22. In the current paper, we present data on the adherence to WCRF/AICR recommendations and data regarding HRQL of the subsequent measurement in January 2014. Patients with unverifiable addresses, with cognitive impairment, who died prior to the start of the study or were terminally ill, with stage 0/carcinoma in situ and those already included in our 2009 CRC study or another study (*n* = 169), were excluded [14]. One thousand six hundred twenty-five participants were invited for the data collection in August 2013, see Fig. 1. Between August 2013 and January 2014, 78 (4.8%) participants died or discontinued participation, resulting in 1547 survivors who were invited for the questionnaire on HRQL in January 2014. Figure 1 gives an overview of the number of non-responders and excluded patients. Of the 1625 CRC survivors who were invited in August 2013, 1096 were included in the present study (67.4% of invited participants in August 2013) (Fig. 1).

### Health-related quality of life

The validated European Organization for Research and Treatment of Cancer–Quality of Life Questionnaire (EORTC QLQ)-C30 was used to assess HRQL and fatigue [15, 16]. For CRC patients, previous research concludes that a healthy lifestyle is mainly associated with functioning scales (i.e. physical, emotional, social, cognitive and role functioning) and fatigue [5, 17]. Therefore, only functioning scales, fatigue and global health status were included in the analysis. All items were scored on a 4-point Likert scale ranging from ‘not at all’ to ‘very much’, except for the items regarding global health status which were scored from 1 (very poor) to 7 (excellent). All scores were linearly transformed to a scale ranging from 0 to 100 points [15, 18]. Higher scores on functioning scales represent better functioning, while a higher score on the fatigue scale corresponds to more fatigue.

Changes in scores were considered clinically relevant if the mean difference was 5–14 points for physical functioning, 5–11 points for social functioning, 3–9 points for cognitive functioning, 6–19 points for role functioning and 5–13 point for fatigue [19]. For emotional functioning no cut-offs were defined [19].

### Adherence to the WCRF/AICR recommendations

Adherence to the eight WCRF/AICR recommendations was determined, six recommendations about healthy diet, one about body fatness and one about physical activity. The scoring of adherence to the WCRF/AICR recommendations is described extensively by Winkels et al. [20] and Romaguera et al. [21].

With regard to a healthy diet, in 2007, the WCRF/AICR published the following recommendations: ‘foods and drinks that promote weight gain: avoid high-calorie foods and sugary drinks’, ‘plant-based foods: eat more grains, vegetables, fruit and beans’, ‘animal foods: limit red meat and avoid processed meat’, ‘alcoholic drinks: for cancer prevention, don’t drink alcohol’, ‘preservation, processing & preparation: eat less salt and avoid mouldy grains & cereals’ and ‘dietary supplement use: for cancer prevention, don’t rely on supplements’. To assess adherence to the recommendations concerning healthy diet, the Dutch Healthy Diet-Food Frequency Questionnaire (DHD-FFQ) was used [22]. The original DHD-FFQ consists of 34 items. To compensate for items that were missing in the DHD-FFQ but are incorporated in the WCRF/AICR recommendations, additional questions on intake of meat, processed meat and sugary beverages were added to the questionnaire, from now on called WCRF/DHD-FFQ. The WCRF/DHD-FFQ consists of 40 items on intakes of bread, fruit, vegetable, potatoes, milk, cheese, meat products, fish, cookies, pastries, crisps, soup, fats and oils, take-away food, pizza, sugary drinks, alcoholic beverages and discretionary salt.

Adherence to the recommendation regarding body fatness was determined based on body mass index (BMI) by calculating weight (kg)/height (m)^2^. Weight and height were self-reported. BMI was categorised as normal weight (18.5 < BMI < 25 kg/m^2^), overweight (25 > BMI < 30 kg/m^2^) or obesity (BMI > 30 kg/m^2^).

Physical activity was assessed using the Short Questionnaire to Assess Health-Enhancing Physical Activity (SQUASH) which contains questions about multiple activities referring to a normal week in the past month. Results were converted to time spent in light, moderate and vigorous activities, which were then converted to activity scores [23]. When this total activity score was 5 or more, representing the number of activities of at least 30 min per week, persons were categorised as adherent to the physical activity recommendation. If one of the recommendations was met, participants received 1 point for that recommendation.

When a recommendation was not met, 0 or 0.5 points were allotted according to the available cut-off values. The total score had a range of 0–8; a higher score means better adherence to the recommendations [20].

### Analysis and statistical methods

Responders were compared to non-responders. The CRC survivors were categorised into three groups, based on tertiles of WCRF/AICR adherence scores following the sample distribution. Chi-square (categorical variables) and one-way ANOVA (continuous variables) were used to test for differences in baseline characteristics.

To assess the association between WCRF adherence scores and HRQL, linear regression models were used both for the tertiles and for the continuous adherence scores. The following variables were tested whether they changed the regression coefficient by at least 10% [24]: gender, age, comorbidities, smoking status, years since diagnosis, tumour localization, tumour stage, having a stoma, chemotherapy and radiotherapy; and for the analyses of the individual components diet, physical activity and BMI. Gender (male/female), age (continuous), comorbidities (no comorbidities, 1 comorbidity, > 2 comorbidities) and smoking (current, former, never) changed the regression coefficient ≥ 10% and were included in the multivariable model. For the analyses of the individual components, diet and BMI changed by > 10% when physical activity was added to the model, therefore physical activity was added to the multivariable model. Dummy variables were created for WCRF/AICR adherence score tertiles, smoking status and comorbidities.

Functioning scales, global health status and fatigue were also examined separately in relation to each of the three components of the adherence score (BMI (normal weight, overweight and obese), physical activity (score 0/1) and diet (low adherence (score < 3 points), moderate adherence (score 3–< 4 points) and high adherence (> 4 points)). To evaluate the effect of the separate components of adherence scores on the functioning scales, global health status and fatigue beyond the effects of the other components, the analysis of each component was adjusted for the other components.

A *p* value < 0.05 was regarded as statistically significant. All analyses were conducted using the Statistical Package for Social Sciences (SPSS) version 23.0 (IBM).

## Results

### General characteristics of the study population

Respondents of our study were most often male, > 65 years old, had two or more comorbidities, were former smokers, had a mean time since diagnosis of 8.1 years, had a colon tumour, stage II, and did not receive chemotherapy or radiotherapy (Table 1). When comparing the non-respondents and excluded respondents to the included respondents, non-respondents and excluded respondents did not differ from respondents (data not shown).

**Table 1:** Sociodemographic and clinical characteristics for the three tertiles of WCRF/AICR adherence scores (*N* = 1096)

		Total population	Tertile 1 WCRF adherence score < 4.42 points	Tertile 2 WCRF adherence score 4.42–5.33 points	Tertile 3 WCRF adherence score >5.33 points
		*N* (%)	*N* (%)	*N* (%)	*N* (%)
*N*	1096 (100%)	360 (33%)	365 (33%)	371 (34%)	
Gender*					
	Male	635 (58%)	227 (63%)	229 (63%)	179 (48%)
	Female	461 (42%)	133 (37%)	136 (37%)	192 (52%)
	Missing	0	0	0	0
Age*	Mean age (years + SD)	70.8 + 9.2	69.7 + 9.5	70.9 + 9.1	71.7 + 8.9
	< 65 years	264 (24%)	102 (28%)	89 (24%)	73 (20%)
	> 65 years	832 (76%)	258 (72%)	276 (76%)	298 (80%)
	Missing	0	0	0	0
Comorbidities					
	0	261 (24%)	76 (21%)	85 (23%)	100 (27%)
	1	306 (28%)	96 (27%)	102 (28%)	108 (29%)
	> 2	495 (45%)	183 (51%)	163 (45%)	149 (40%)
	Missing	34 (3%)	5 (1%)	15 (4%)	14 (4%)
Smoking*					
	Current	85 (8%)	33 (9%)	26 (7%)	26 (7%)
	Former	667 (61%)	237 (66%)	224 (61%)	206 (56%)
	Never	322 (29%)	83 (23%)	106 (29%)	133 (36%)
	Missing	22 (2%)	7 (2%)	9 (3%)	6 (2%)
Years since diagnosis					
	Mean time since diagnosis (SD)	8.1 + 2.8	8.1 + 2.8	8.2 + 2.8	7.9 + 2.8
	< 5 years	116 (11%)	39 (11%)	32 (9%)	45 (12%)
	> 5 years	980 (89%)	321 (89%)	333 (30%)	326 (88%)
	Missing	0	0	0	0
Tumour localization					
	Colon	634 (58%)	204 (57%)	211 (58%)	219 (59%)
	Rectum	462 (42%)	156 (43%)	154 (42%)	152 (41%)
	Missing	0	0	0	0
Tumour stage					
	Stage I	348 (32%)	111 (31%)	112 (31%)	125 (34%)
	Stage II	372 (34%)	108 (30%)	129 (35%)	135 (37%)
	Stage III	318 (29%)	119 (33%)	101 (28%)	98 (26%)
	Stage IV	26 (2%)	10 (3%)	12 (3%)	4 (1%)
	Missing	31 (3%)	12 (3%)	11 (3%)	8 (2%)
Stoma					
	Yes	168 (15%)	58 (16%)	46 (13%)	64 (17%)
	No	928 (85%)	302 (84%)	319 (87%)	307 (83%)
	Missing	0	0	0	0
Chemotherapy*					
	Yes	329 (30%)	128 (36%)	113 (31%)	88 (24%)
	No	767 (70%)	232 (64%)	252 (69%)	283 (76%)
	Missing	0	0	0	0
Radiotherapy					
	Yes	370 (34%)	125 (35%)	119 (33%)	126 (34%)
	No	726 (66%)	235 (65%)	246 (67%)	245 (66%)
	Missing	0	0	0	0

*p < 0.05

The mean total WCRF/AICR adherence score was 4.81 ± 1.04 of a total of 8 points (range 1.33–8.00).

Higher WCRF/AICR adherence scores were more common among women compared to men. The highest WCRF/AICR adherence scores were found among survivors who never smoked, among older participants and among participants who did not receive chemotherapy (Table 1). Years since diagnosis, tumour localization and stage, having a stoma, comorbidities and receiving radiotherapy, were evenly distributed among the tertiles of WCRF/AICR adherence scores. Thirty-four percent of respondents adhered to the BMI recommendation: ‘maintain body weight within the normal range from age 21; BMI 18.5 <25 kg/m^2^, 75% adhered to the physical activity recommendation: ‘be moderately physically active, equivalent to brisk walking, for at least 30 min every day’ and the mean dietary adherence score was 3.48 + 0.87 of a total of 6 points (range 0.5–6.0). Fifty-eight percent adhered to the recommendation ‘foods and drinks that promote weight gain: avoid sugary drinks’ with adherence = no sugary drinks, 10% adhered to ‘plant-based foods: at least five portions/servings (at least 400 g) of a variety of non- starchy vegetables of fruits every day’ with adherence = a mean fruit and vegetable intake > 400 g/day and dietary fibre > 17 g/day, 8% to ‘meat products: people who eat red meat to consume less than 500 g/week, very little, if any, to be processed’ with adherence = red/processed meat < 500 g/week of which processed meat < 3 g/day, 73% to ‘alcoholic drinks: If alcoholic drinks are consumed, limit consumption to no more than two drinks a day for men, and one drink a day for women’, 12% to ‘preservation, processing & preparation: Limit consumption of processed foods with added salt to ensure an intake of <6 g (2.4 g sodium) a day’ and 75% to the recommendation on ‘dietary supplement use: Dietary supplements are not recommended for cancer prevention’.

Higher HRQL scores were seen among men (physical, role, emotional functioning and global health status), for younger participants (< 65 years old) (physical and emotional functioning), CRC survivors without comorbid conditions (for physical, role, emotional, cognitive, social functioning and global health status and fatigue) and those who never smoked (emotional functioning and global health status). For years since diagnosis, tumour localization, tumour stage, having a stoma, receiving chemotherapy and radiotherapy, no significant differences in HRQL was found (data not shown).

### Health-Related Quality of Life

**Fig. 2:**
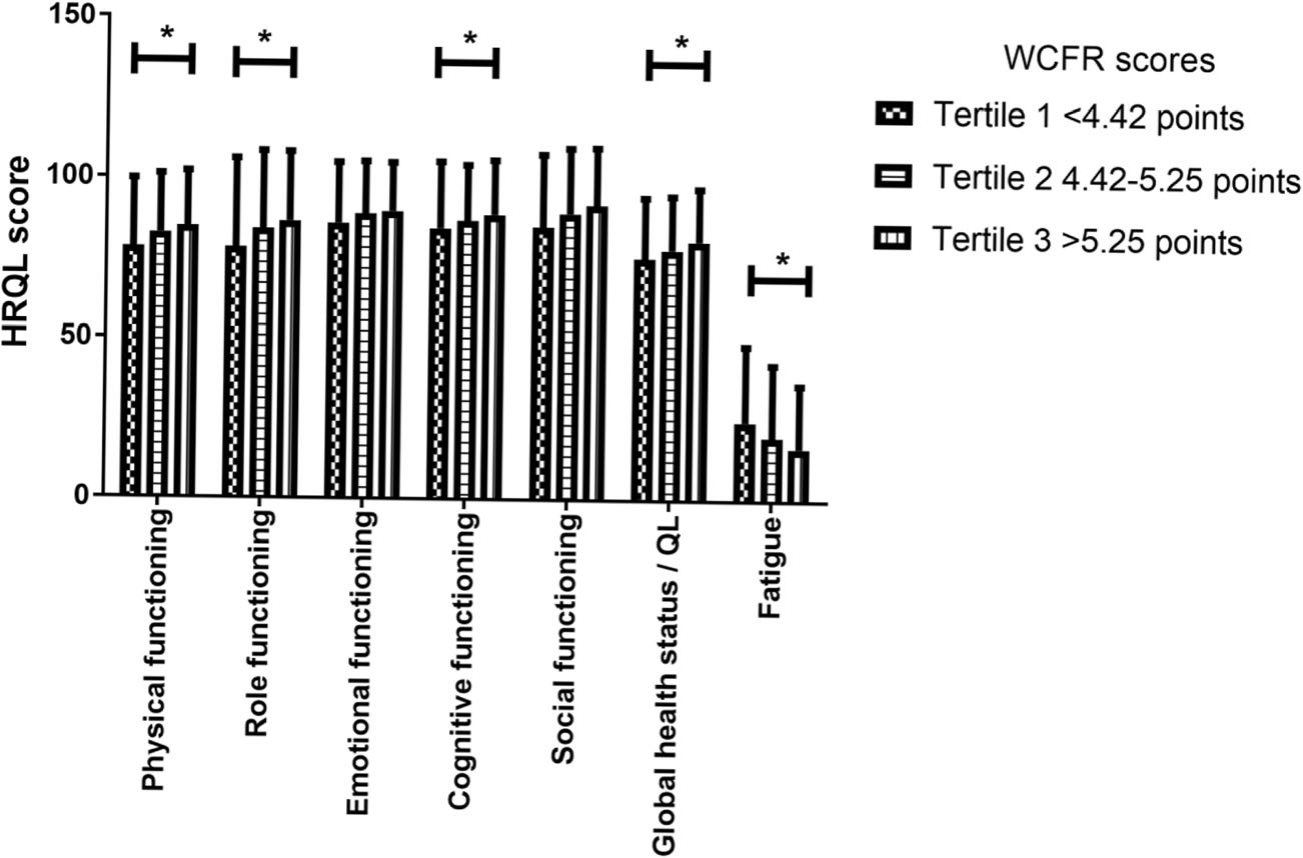
HRQL scores by WCRF/AICR adherence scores (*N* = 1096). A single asterisk denotes small clinically relevant difference between tertile 1 and tertile 3

Survivors with the highest WCRF/AICR adherence scores (tertile 3; > 5.25 points) had the highest mean physical functioning scores (84.8 + 17.2 vs. 78.3 + 21.3) and role functioning scores (86.5 + 21.7 vs. 78.3 + 27.7) and the lowest mean scores on fatigue (16.6 + 19.7 vs. 24.7 + 23.7), compared to survivors with the lowest WCRF/AICR adherence scores (tertile 1; < 4.42 points; see Fig. 2). Although small, these differences are considered clinically relevant [19].

Multivariable linear regression models (Table 2) showed that compared to the lowest tertile (< 4.42 points), the second (4.42–5.25 points) and the third tertile (> 5.25 points) of the WCRF/AICR adherence score were significantly associated with higher scores on physical, role and social functioning and a lower level of fatigue. The highest tertile of the adherence score was significantly associated with higher scores on emotional functioning, cognitive functioning and global health status compared to the lowest tertile. For an increase in the continuous score of adherence to the WCRF/AICR recommendations, significant associations were found for better physical functioning, role functioning, cognitive functioning, social functioning and global health status and less fatigue.

**Table 2:** The association between overall WCRF/AICR adherence score and HRQL and fatigue using multivariable linear regression (*N* = 1096)

HRQL	WCRF adherence scores			
	Tertile 1 < 4.42 points	Tertile 2 4.42–5.25 points	Tertile 3 > 5.25 points	Continuous
Physical functioning	REF	3.88 (4.42, 6.33)*	6.94 (4.46, 9.42)*	2.71 (1.73, 3.68)*
Role functioning	REF	4.76 (1.40, 8.12)*	7.49 (4.09, 10.89)*	2.87 (1.53, 4.21)*
Emotional functioning	REF	2.35 (− 0.06, 4.75)	3.34 (0.90, 5.77)*	0.85 (− 0.11, 1.81)
Cognitive functioning	REF	1.90 (− 0.77, 4.57)	3.48 (0.78, 6.17)*	1.25 (0.19, 2.32)*
Social functioning	REF	3.56 (0.67, 6.44)*	6.12 (3.21, 9.04)*	2.01 (0.85, 3.16)*
Global health status/QL	REF	1.68 (− 0.70, 4.07)	4.33 (1.92, 6.74)*	1.64 (0.69, 2.59)*
fatigue	REF	− 3.87 (− 6.90, − 0.84)*	− 7.65 (− 10.72, − 4.59)*	− 2.81 (− 4.02, − 1.60)*

Results are expressed as *β* (95% confidence interval (CI)). All models were adjusted for age, gender, comorbidities and smoking. An increase in functioning scores and global health status indicates an improvement in HRQL. A decrease in fatigue scores indicates an improvement in fatigue

*p < 0.05

Multivariable linear regression models showed that adherence to the physical activity recommendation was associated with better physical, role, emotional and social functioning, better global health status and less fatigue (Table 3). Being overweight was not significantly associated with different HRQL and fatigue scores compared to participants with a healthy weight. However, being obese was significantly associated with lower physical functioning compared to healthy weight respondents. Adherence to the dietary recommendations was not associated with the different functioning scales, global health status or fatigue.

**Table 3:** The association between HRQL and fatigue and physical activity, diet and BMI using multivariable linear regression (*N* = 1096)

		Physical functioning	Role functioning	Emotional functioning	Cognitive functioning	Social functioning	Global health status/QL	Fatigue
Physical activity								
	0	REF	REF	REF	REF	REF	REF	REF
	1	10.30 (8.01; 12.59)*	10.50 (7.31; 13.67)*	2.75 (0.45; 5.06)*	2.49 (− 0.07; 5.04)	6.20 (3.45; 8.95)*	6.27 (4.02; 8.53)*	− 7.43 (− 10.32; − 4.54)*
Diet								
	0– < 3	REF	REF	REF	REF	REF	REF	REF
	3– < 4	0.42 (− 1.98; 2.82)	1.55 (− 1.79; 4.89)	1.05 (− 1.38; 3.47)	1.52 (− 1.53; 3.83)	1.64 (− 1.25; 4.54)	1.96 (− 0.41; 4.33)	− 2.87 (− 5.91; 0.16)
	> 4	0.09 (− 2.45; 2.64)	0.38 (− 3.17; 3.92)	0.39 (− 2.18; 2.96)	1.71 (− 1.14; 4.55)	0.70 (− 2.37; 3.77)	0.26 (− 2.25; 2.78)	− 3.12 (− 6.34; 0.10)
BMI								
	Normal weight	REF	REF	REF	REF	REF	REF	REF
	Overweight	− 0.24 (− 2.46; 1.98)	0.29 (− 2.80; 3.37)	0.97 (− 1.27; 3.20)	− 0.58 (− 3.06; 1.90)	0.22 (− 2.45; 2.89)	1.74 (− 0.45; 3.93)	− 0.49 (− 3.23; 2.32)
	Obese	− 4.15 (− 7.16; − 1.15)*	1.73 (− 5.91; 2.46)	− 0.92 (− 3.95; 2.11)	− 2.80 (− 6.15; 0.55)	− 1.93 (− 5.54; 1.69)	− 0.29 (− 3.25; 2.67)	2.81 (− 0.98; 6.60)

Results are expressed as *β* (95% confidence interval (CI)). All models were adjusted for age, gender, comorbidities and smoking. An increase in functioning scores and global health status indicates an improvement in HRQL. A decrease in fatigue scores indicates an improvement in fatigue

*p < 0.05

## Discussion

Higher adherence to the WCRF/AICR recommendations was associated with better physical functioning, role functioning, social functioning and global health status and less fatigue among CRC survivors. Physical activity seemed to be the main component of the WCRF/AICR recommendations contributing to the observed associations. Being obese was associated with worse physical functioning. Diet was not associated with the different functioning scales, global health status and fatigue.

Previous studies showed an association between higher adherence to the non-cancer-specific Healthy Eating Index or the Mediterranean diet and higher levels of HRQL in cancer survivors, including CRC survivors [5–7]. Our study did not show an association between the specific dietary recommendations of the WCRF/AICR and HRQL, however when looking at the total adherence WCRF/AICR recommendations score, an association between level of adherence and HRQL was found in CRC survivors similar to the association with the Healthy Eating Index or the Mediterranean diet [5–7]. Our study only found an inverse association between being obese and physical functioning. Our findings are in line with the results of Inoue-Choi who showed that higher adherence to the WCRF/AICR recommendations, especially to the physical activity recommendations, was significantly associated with higher physical and mental component summary scores (SF-36) in a population of female cancer survivors with different cancer types [10]. Our results are also in line with the results of Breedveld-Peters et al. who found that higher adherence to the total set of WCRF/AICR recommendations was associated with better physical functioning and less fatigue in a small group (*N* = 145) of CRC survivors in the Netherlands [12].

Of all recommendations, physical activity was most strongly associated with most functioning scales: physical, role, emotional and social functioning; global health status and fatigue in our study. When investigating the crude model, diet was associated with physical functioning. However, when we adjusted for physical activity, as discussed in the “Subjects and Methods” section, the association was no longer significant. This indicates that physical activity indeed was the main component of the WCRF/AICR associated with a better HRQL and not diet.

For fatigue, there is ample evidence that physical activity has a positive influence [25]. This is also in line with the US National Comprehensive Cancer Network guidelines for managing fatigue [26]. Two observational studies recommended that CRC survivors should meet the public health exercise guideline (> 150 min of moderate to strenuous intensity exercise or > 60 min of strenuous intensity exercise per week), since CRC survivors who meet these standards had a higher quality of life than other survivors who did not meet these exercise guidelines [8, 14]. However, to be able to be physically active, a healthy diet and body weight are important. This is supported by our finding that being obese was negatively associated with physical functioning. Therefore, it remains important to focus on the triad of physical activity, diet and body weight when targeting CRC survivors, as was also suggested by Blanchard et al. [27].

The differences found in functioning scales when comparing respondents with the highest WCRF/AICR adherence scores (> 5.25) to those with the lowest scores (< 4.42) are subtle but nevertheless clinically meaningful, meaning that these differences are noticeable in daily clinical practice [19].

The present study was the first study investigating the associations between adherence to the cancer-specific WCRF/AICR recommendations and HRQL, in a large group of male and female CRC survivors. Major strengths of our study are the large sample size that made it possible to investigate the association between diet, physical activity and body fatness on HRQL separately, and the registry-based character of the study. However, there are also some limitations. First, selection bias may limit the generalizability of our findings. The present study covers CRC stage I–IV, with a mean time since diagnosis of 8.1 years. Patients with a worse prognosis or worse health might be less likely to participate in the study, might have more problems to complete the follow-up questionnaires or might have already died. Although our included participants were older and more often former smokers than the excluded participants, our study population may consist of more healthy CRC survivors possibly with different associations between a healthy lifestyle and HRQL. Absolute HRQL scores of our CRC survivors should be interpreted cautiously and are not generalizable for the whole population of CRC survivors. Second, data in our study were self-reported by survivors, which might have led to underreporting (body weight) and overreporting (physical activity, consumption of vegetable and fruits) due to social desirable answers [28–31]. Self-reporting of nutritional intake may lead to differential misclassification: obese participants might underreport their intake more than non-obese participants and elderly might be more eager to present themselves in a favourable way, giving a social desirable answer, hence their higher WCRF/AICR scores 32. Also non-differential misclassification might have occurred, probably leading to higher WCRF/AICR adherence scores. However, only one respondent had a total score of 8 points and the percentage survivors adhering to specific recommendations e.g., more plant-based foods, less red meat was often low, demonstrating how hard it is for CRC survivors and for the general public to adhere to the WCRF/AICR recommendations. Third, 75% of respondents reported to meet the guidelines for physical activity. This might be an overestimation, due to the nature of measuring physical activity: by means of a questionnaire (SQUASH) and not by the use of activity trackers. Fourth, the scoring of fatigue and other functioning scales by use of the EORTC QLQ-C30 may not be the best way to determine HRQL, especially fatigue, so many years after treatment. However, the EORTC QLQ-C30 is the most common questionnaire to determine HRQL in cancer survivors. Hence, it makes it easy to compare our results to the work of others.

Finally, due to the study design, with 6 months between the questionnaire on adherence to the WCRF recommendations and the questionnaire on HRQL in a cohort with a mean time since diagnosis of 8.1 years, we cannot draw conclusions whether the association between HRQL and adherence to the WCRF/AICR recommendations reflects a causal relation or reverse causation which means that survivors who have a better HRQL easier adhere to the WCRF/AICR recommendations.

## Conclusion

Higher adherence to the WCRF/AICR recommendations was associated with better physical, role, cognitive and social functioning, better global health status and less fatigue among CRC survivors. Physical activity seemed to be the main contributor to higher scores on most functioning scales and global health status and lower scores on fatigue in CRC survivors.

Because CRC survivors with the highest adherence to the WCRF/AICR recommendations also report the highest HRQL, we recommend to investigate whether increasing the adherence in CRC survivors indeed results in better HRQL. However, previous research as well as the present study has shown that it is very difficult to motivate cancer survivors to positively change their lifestyle [12, 20]. Even Lynch syndrome carriers, with a very high inherited risk of developing CRC [33], from whom we hoped that they would be extremely motivated to change their lifestyle, were shown to adhere to those recommendations only in a slightly better manner than CRC survivors without Lynch syndrome. Adhering to the WCRF/AICR recommendations can be challenging for CRC survivors. Thus, trials aiming to increase adherence should not only focus on the effects better adherence has on cancer outcomes but also on tools to stimulate and motivate CRC survivors to follow the recommendations to the best of their abilities.
